# Imbalance of Microbacterial Diversity Is Associated with Functional Prognosis of Stroke

**DOI:** 10.1155/2023/6297653

**Published:** 2023-05-08

**Authors:** Xintong Zhang, Xiangyu Wang, Hong Zhao, Risheng Cao, Yini Dang, Binbin Yu

**Affiliations:** ^1^Department of Rehabilitation Medicine, The First Affiliated Hospital of Nanjing Medical University, Jiangsu, China; ^2^Department of Rehabilitation Medicine, The Affiliated Lianyungang Oriental Hospital of Kangda College of Nanjing Medical University, Jiangsu, China; ^3^Department of Science and Technology, The First Affiliated Hospital of Nanjing Medical University, Jiangsu, China; ^4^Department of Gastroenterology, The First Affiliated Hospital of Nanjing Medical University, Jiangsu, China

## Abstract

**Objectives:**

There is mounting evidence to suggest that the pathophysiology of stroke is greatly influenced by the microbiota of the gut and its metabolites, in particular short-chain fatty acids (SCFAs). The primary purpose of the study was to evaluate whether the levels of SCFAs and the gut microbiota are altered in poststroke patients and to examine the relationship between these alterations and the physical condition, intestinal health, pain, or nutritional status of patients.

**Methods:**

Twenty stroke patients and twenty healthy controls were enrolled in the current study, and their demographics were matched. Gas chromatography was used to determine the fecal SCFAs, and 16S rRNA gene sequencing was used to evaluate their fecal microbiota. Microbial diversity and richness were examined using the diversity indices alpha and beta, and taxonomic analysis was utilized to determine group differences. The relationships between the gut microbiome and fecal SCFAs, discriminant bacteria, and poststroke clinical outcomes were analyzed.

**Results:**

Less community richness (ACE and Chao) was observed in the poststroke patients (*P* < 0.05), but the differences between the poststroke group and the healthy control group in terms of species diversity (Shannon and Simpson) were not statistically significant. The makeup of the poststroke gut microbiota was distinct from that of the control group, as evidenced by beta diversity. Then, the relative abundances of the taxa in the poststroke and control groups were compared in order to identify the specific microbiota changes. At the level of phylum, the poststroke subjects showed a significant increase in the relative abundances of *Akkermansiaceae*, *Fusobacteriota*, *Desulfobacterota, Ruminococcaceae*, and *Oscillospirales* and a particularly noticeable decrease in the relative abundance of *Acidobacteriota* compared to the control subjects (*P* < 0.05). In regard to SCFA concentrations, lower levels of fecal acetic acid (*P* = 0.001) and propionic acid (*P* = 0.049) were found in poststroke subjects. *Agathobacter* was highly correlated with acetic acid level (*r* = 0.473, *P* = 0.002), whereas *Fusobacteria* (*r* = −0.371, *P* = 0.018), *Flavonifractor* (*r* = −0.334, *P* = 0.034), *Desulfovibrio* (*r* = −0.362, *P* = 0.018), and *Akkermansia* (*r* = −0.321, *P* = 0.043) were negatively related to acetic acid levels. Additionally, the findings of the correlation analysis revealed that *Akkermansia* (*r* = −0.356, *P* = 0.024), *Desulfovibrio* (*r* = −0.316, P = 0.047), and *Alloprevotella* (*r* = −0.366, *P* = 0.020) were significantly negatively correlated with high-density lipoprotein cholesterol. In addition, the Neurogenic Bowel Dysfunction score (*r* = 0.495, *P* = 0.026), Barthel index (*r* = −0.531, *P* = 0.015), Fugl-Meyer Assessment score (*r* = −0.565, *P* = 0.009), Visual Analogue Scale score (*r* = 0.605, P = 0.005), and Brief Pain Inventory score (*r* = 0.507, *P* = 0.023) were significantly associated with alterations of distinctive gut microbiota.

**Conclusions:**

Stroke generates extensive and substantial alterations in the gut microbiota and SCFAs, according to our findings. The differences of intestinal flora and lower fecal SCFA levels are closely related to the physical function, intestinal function, pain, or nutritional status of poststroke patients. Treatment strategies aimed at modulating the gut microbiota and SCFAs may have the potential to enhance the clinical results of patients.

## 1. Introduction

Stroke is the main cause of disability and death, respectively, and imposes huge individual and societal burdens [1, 2]. Although advanced stroke emergency treatments, such as endovascular thrombectomy and intravenous thrombolysis, can improve the physical and mental status of some patients, the prognosis of most stroke patients is still poor [3].

Recent studies have focused on the finding of the microbiome-gut-brain axis, which describes the relationship between the gut and the brain via gut bacteria [4]. The microbiome-gut-brain axis consists primarily of gut microbiota and its metabolites, neurological (enteric, central, and autonomic nervous systems), immunological, and hormonal pathways, of which gut microbiota is an important component [5, 6]. Stroke is commonly associated with hypertension, diabetes, hyperlipidemia, and low physical activity, all of which have major influences on the gut microbiota [7]. In addition, stressful stimuli at the onset of stroke, limb paralysis, neurogenic intestinal dysfunction, neuropathic pain, malnutrition, and other problems caused by stroke will lead to microbiome disturbances [3, 8, 9]. On the other hand, the gut microbiota and its metabolites, such as the highly concerned short-chain fatty acids (SCFAs), may affect poststroke outcomes through multiple pathways, including intestinal leakage, local and systemic inflammation, and endotoxemia [10]. The gut microbiota and its metabolites have great potential to become therapeutic targets for stroke.

Some studies have demonstrated the existence of significant intestinal flora disturbance in poststroke patients [11, 12]. Our previous study also found that stroke may lead to changes in gut microbiota structure, especially a significant decrease in the abundance of SCFA-producing microbiota, but the level of SCFAs was not explored in that study [13]. A recent study reported that reduced SCFAs, especially acetate, were associated with poor motor functional outcomes after stroke [14]. However, that study did not explore the relationship between SCFAs and other complications, such as gastrointestinal dysfunction, pain, and malnutrition. These complications may have potential interactions with intestinal flora and SCFAs, which are also important factors affecting the long-term prognosis of stroke patients [9, 15–17].

We carried out this research to evaluate the following two hypotheses by comparing the gut microbiota composition and SCFA levels of poststroke patients with those of healthy individuals: (1) the makeup of the gut microbiota and levels of SCFAs in poststroke patients differ significantly from those of healthy controls, and (2) the alteration of gut microbiota composition and SCFA level in poststroke patients may be potentially related to physical function, intestinal function, pain, and nutritional status.

## 2. Methods and Materials

### 2.1. Study Design and Patient Enrollment

An individual-center prospective observational case-control research was conducted. Patients were recruited from the regular medical wards or the stroke unit at the Affiliated Lianyungang Oriental Hospital of Kangda College of Nanjing Medical University from 19 January 2022 to 29 July 2022. The inclusion criteria were as follows: (1) age of between 18 years and 80 years, (2) ischemic/hemorrhagic stroke as confirmed by computerised tomography (CT) or magnetic resonance imaging (MRI), and (3) were able to provide a vocal response to the directions they were given and provided informed consent [18]. Patients were excluded from the study if (1) diagnosed with silent cerebral infarction or transient ischemic attack (TIA), (2) with serious cognitive impairments or mental dysfunctions, and (3) current participation in another clinical trial or participation in another clinical trial in the 6 months prior to enrolment [19]. Age-, gender-, and risk factor-matching healthy subjects served as the controls. Prior to conducting the study, ethics approval using an approval code was acquired (Institutional Review Board, 2022-041-01). The clinical trial was formally registered in advance with the Clinical Trials Registry (registration number: NCT03938311). Prior to enrolment, consent was acquired with knowledge.

### 2.2. Clinical Assessment and Sample Collection

The following demographic data was collected: age, gender, and subtype of stroke. Clinical assessments were conducted by a trained researcher. The degree of physical symptoms, such as pain, was assessed using tools such as the Visual Analogue Scale (VAS) as well as the Brief Pain Inventory (BPI). A VAS value of 0 showed that there was no pain, while a VAS score of 10 indicated severe pain [20, 21]. The BPI was used to characterize pain severity and functional interference in daily life. On a scale from 0 (never interferes) to 10 (totally interferes), participants evaluate each item [22, 23]. Bowel function was assessed by using the Neurogenic Bowel Dysfunction (NBD) score, for which a higher score indicates worse bowel function [24, 25]. Scores on the Barthel index (BI) range from 0 to 100, with higher scores showing better performance in activities of daily living (ADL) [26, 27]. The Fugl-Meyer Assessment, often known as the FMA score, was used to evaluate either the upper or lower extremity motor function, and higher score represents better function [28, 29]. Patients' fresh stool samples were taken and stored at a temperature of -80 degrees Celsius for use in DNA extraction at a later time.

### 2.3. DNA Extraction, 16S rRNA Gene Amplification, and Sequencing

Using the Qiagen QIAamp DNA Stool Mini Kit (Qiagen, catalogue number 51504, Hilden, Germany) and following the manufacturer's instructions, bacterial genomic DNA was extracted from the prepared frozen cecal samples. The DNA concentration and purity were evaluated both with a NanoDrop-2000 spectrophotometer (NanoDrop Technologies, Wilmington, DE, USA). For the microbial community diversity analysis, the V3-V4 region of the bacterial 16S rRNA gene was targeted with the barcoded primer pair 341F/806R (341F: CCTAYGGGRBGCASCAG, 806R: GGACTCNNGGGTATCTAAT). The Illumina 16S Metagenomic Sequencing Library preparation protocol was followed to perform the 16S rRNA gene amplification and index PCR for sequencing (Illumina, San Diego, CA, USA).

### 2.4. Quantification of SCFAs in Stool Samples

According to other reports, gas chromatography-mass spectrometry (GC-MS) was used to quantify numerous SCFAs (acetic acids, butyric acids, propionic acids, caproic acids, isobutyric acids, isovaleric acids, and valeric acids) in fecal samples [30].

### 2.5. Bioinformatic Gut Microbiota Analyses

Using QIIME v.1.9.1 (QIIME permits analysis of high-throughput community sequencing data) and USEARCH v.10.0, the 16S rRNA gene sequences were processed in this investigation (Magnit search and clustering orders). The raw FASTQ files had their quality filtered by Trimmomatic, and then, USEARCH merged them based on the following criteria: the removal of barcodes and primers, the removal of low-quality reads, and the detection of nonredundancy readings. Sequences assigned by the UPARSE software to the same operational taxonomic units (OTUs) had a 97% similarity rate (version 7.0.1001). With the QIIME software displayed, alpha diversity indices such as ACE, Chao, Shannon, and Simpson were computed, and beta diversity was evaluated using principal coordinate analysis (PCA) and nonmetric multidimensional scaling (NMDS). Linear discriminant analysis (LDA) and linear discriminant effect size (LEfSe) techniques were used to assess metagenomic biomarkers among groups utilizing the Galaxy Online Analysis Platform.

### 2.6. Statistical Analysis

The means and standard deviations of continuous variables are shown. The categorical variables are represented by numbers (percentages). Microbiota data and SCFA levels were tested by one-way analysis of variance (ANOVA) and the Wilcoxon rank-sum test. Alpha diversity and beta diversity among groups were tested by the Wilcoxon rank-sum test. Using the Bonferroni correction, the *P* values were adjusted for multiple testing. Pearson correlation was used to estimate the correlations between bacterial or SCFA levels and clinical evaluations. *P* values under 0.05 were used to determine whether a difference between groups was significant. With SPSS 24.0, all statistical evaluations were completed (SIBM SPSS, Armonk, NY, USA). Software called GraphPad Prism 5.0 was used to plot the data (La Jolla, CA, USA).

## 3. Results

### 3.1. Participant Demographics

Twenty patients with a clinical diagnosis of stroke were evaluated (average age 64 ± 13 years; gender, male : female 11 : 9) and were recruited. In the meantime, 20 healthy persons of the same age and gender were examined (average age 60 ± 8 years; gender, male : female 6 : 14) who attended annual physical examinations and were also recruited. The clinical features and demographics of stroke patients and controls are shown in Table 1.

### 3.2. Poststroke Subjects Harbor an Altered Gut Microbiota Composition

**Figure 1:**
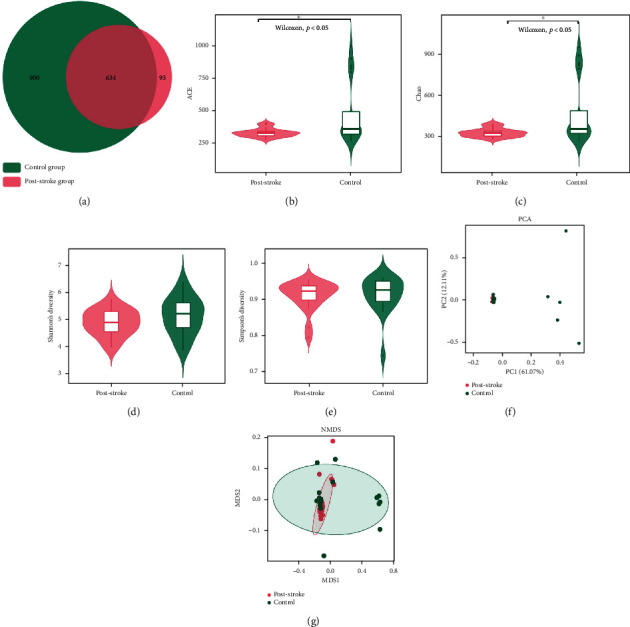
Gut microbiota diversity in poststroke and control subjects. (a) Venn diagram of common OTUs. (b–e) Alpha diversity at the OTU level as measured by the ACE (b), Chao (c), Shannon (d), and Simpson (e) index. (f, g) Beta diversity shown by PCA (f) and NMDS (g) based on weighted UniFrac distance. OTU: operational taxonomic unit; PCA: principal component analysis; NMDS: nonmetric multidimensional scaling.

As shown in Figure 1(a), 900 and 93 OTUs were individually identified from the control group and the poststroke group, and there were 634 OTUs that overlapped between the two groups. Between the poststroke and control groups, there were significant differences (*P* < 0.05) in terms of community richness (ACE and Chao) when comparing bacterial alpha diversity (Figures 1(b) and 1(c)).The differences between each group were not statistically significant when assessing the species diversity of the microbiota (Shannon and Simpson) (Figures 1(d) and 1(e)). PCA and NMDS were used to determine differences in bacterial community composition between the two groups. Poststroke samples were predominantly dissimilar from those of healthy controls, indicating variations in the community structure of the microbiota between the two groups (Figures 1(f) and 1(g)).

**Figure 2:**
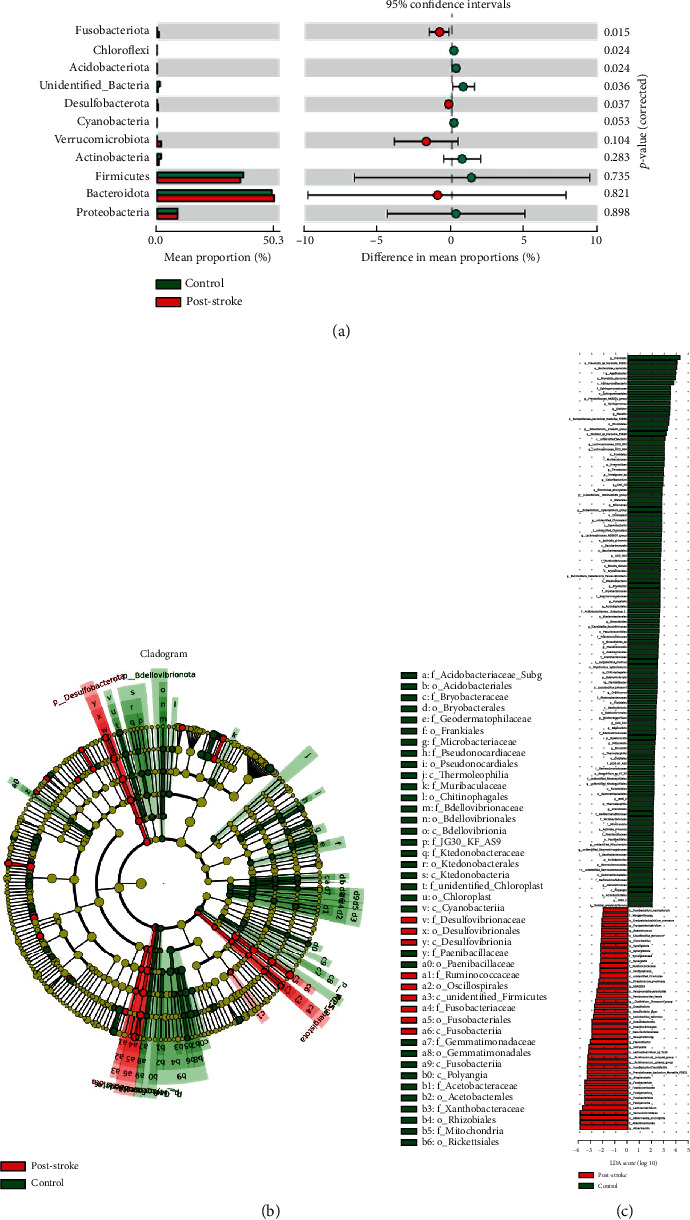
Compositional changes in the gut microbiota of poststroke and healthy controls. (a) The mean relative abundances of taxa at the phylum level in poststroke and control subjects. The red and green bars represent the relative abundances of taxa in poststroke patients and healthy controls, respectively. (b) LEfSe-generated cladograms. (c) LDA scores for the differentially abundant bacterial taxa (LDA score > 2.0). Taxa enriched in the control group are shown by green bars, whereas taxa enriched in the poststroke group are represented by red bars. LEfSe: linear discriminant effect size; LDA: linear discriminant analysis.

We evaluated the average relative abundances of the taxa in the poststroke and control groups to identify the precise changes in the microbiota. At the phylum level, poststroke patients have significantly less *Acidobacteriota* than controls (0.0005% vs. 0.2710%), whereas the abundance of *Fusobacteriota* was considerably increased in poststroke patients (0.9640%) compared to controls (0.0961%). Furthermore, we also observed that *Desulfobacterota* was enriched in poststroke samples compared to control samples (Figure 2(a)). LEfSe was utilized to discover substantial changes in the bacterial composition of the poststroke and control groups. Significantly higher levels of *Akkermansiaceae*, *Fusobacteriota*, *Desulfobacterota*, *Ruminococcaceae*, and *Oscillospirales* were found in the poststroke individuals (Figures 2(b) and 2(c)).

### 3.3. The Levels of SCFAs in the Poststroke Group Differ Significantly from Those of the Control Group

**Figure 3:**
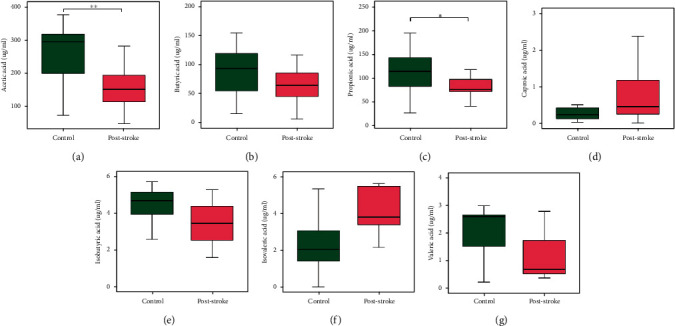
Fecal levels of SCFAs of poststroke and control patients. Boxplots showing the absolute concentration distribution of SCFAs measured in microgram per milliliter in the control group and poststroke group. (a) Acetic acid, (b) butyric acid, (c) propionic acid, (d) caproic acid, (e) isobutyric acid, (f) isovaleric acid, and (g) valeric acid. ^∗^*P* value ≤ 0.05; ^∗∗^*P* value ≤0.01; Wilcoxon rank-sum test. SCFAs: short-chain fatty acids.

In Figure 3, the amounts of acetic acid, butyric acid, propionic acid, caproic acid, isobutyric acid, isovaleric acid, and valeric acid in feces are displayed. The concentration of acetic acid was dramatically reduced in patients with stroke (67.60 ± 36.98) compared with controls (212.28 ± 95.25, *P* = 0.001). Between the two groups, there were no discernible variations in butyric acid levels (*P* = 0.070). Compared with healthy control group (160.41 ± 27.36), the propionate concentration was significantly decreased in the poststroke group (114.54 ± 65.72, *P* = 0.049). However, there were no appreciable variations in the concentrations of caproic acid, isobutyric acid, isovaleric acid, or valeric acid between the groups.

### 3.4. Correlation between the Intestinal Microbiota and Fecal SCFA Levels

**Figure 4:**
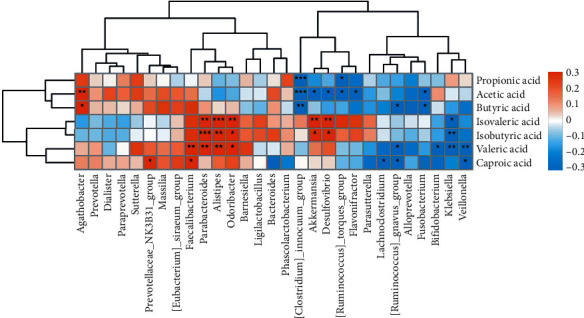
Correlation of the gut microbiota with fecal SCFA levels. SCFA: short-chain fatty acid.

At the genus level, a Pearson correlation was employed to establish a relationship between the differentially abundant taxa and the levels of SCFAs in the feces (shown in Figure 4). The relative abundance of *Agathobacter* was highly correlated with acetic acid level (*r* = 0.473, *P* = 0.002), whereas the relative abundances of *Fusobacteria* (increased considerably in the poststroke group, *r* = −0.371, *P* = 0.018), *Flavonifractor* (*r* = −0.334, *P* = 0.034), *Desulfovibrio* (increased considerably in the poststroke group, *r* = −0.362, *P* = 0.018), and *Akkermansia* (increased considerably in the poststroke group, *r* = −0.321, *P* = 0.043) were negatively correlated with acetic acid level. Furthermore, we discovered a negative association between *Fusobacteria* and butyrate (*r* = −0.362, *P* = 0.022). Additionally, there was a positive correlation between the amounts of isovaleric acid and isobutyric acid and the presence of *Desulfovibrio*, *Akkermansia*, *Parabacteroides*, *Alistipes*, and *Odoribacter*.

### 3.5. Correlations among Fecal SCFA Concentrations, Distinct Bacterial Species, and Clinical Variables

**Figure 5:**
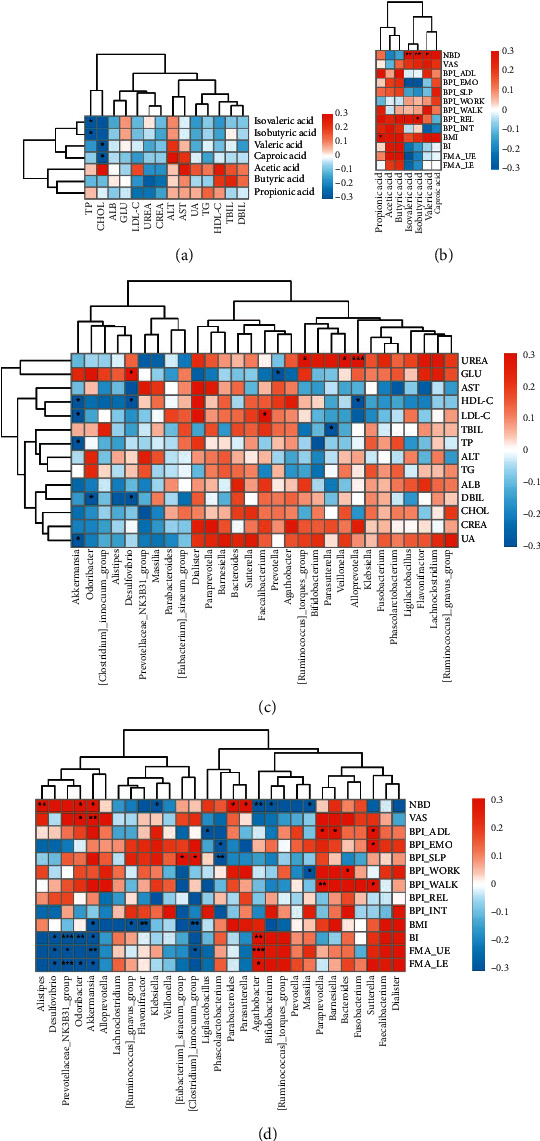
Correlations of fecal SCFA levels with serum index and poststroke clinical variation. (a) Correlation between fecal SCFA levels with serum index; (b) correlation between fecal SCFA levels with poststroke clinical variation; (c) correlation between differentiated bacterial genus with serum index; (d) correlation between differentiated bacterial genus with poststroke clinical variation. SCFA: short-chain fatty acid; DBil: direct bilirubin; TBil: total bilirubin; HDL-C: high-density lipoprotein cholesterol; TG: triglyceride; UA: uric acid; ALT: alanine aminotransferase; AST: aspartate aminotransferase; CREA: creatinine; UREA: urea; LDL-C: low-density lipoprotein cholesterol; GLU: glucose; ALB: albumin; CHOL: cholesterol; TP: total protein; NBD: Neurogenic Bowel Dysfunction; VAS: Visual Analogue Scale; BPI: Brief Pain Inventory; BMI: body mass index; BI: Barthel index; FMA-UE: Fugl-Meyer Assessment Upper Extremity Scale; FMA-LE: Fugl-Meyer Assessment Lower Extremity Scale.

In order to determine whether there are any significant relationships between various clinical indexes, including blood parameters, functional parameters, SCFA levels, and clinical parameters and distinct bacterial species, Pearson correlation analysis was used. Isovaleric acid (*r* = −0.344, *P* = 0.030) and isobutyric acid (*r* = −0.335, *P* = 0.034) were negatively correlated with serum total protein (TP). Valeric acid (*r* = −0.338, *P* = 0.032) and caproic acid (*r* = −0.390, *P* = 0.012) were negatively correlated with cholesterol (Figure 5(a)). Furthermore, isovaleric acid (*r* = 0.636, *P* = 0.003), isobutyric acid (*r* = 0.606, *P* = 0.005), and valeric acid (*r* = 0.456, *P* = 0.043) were positively correlated with NBD (Figure 5(b)).

The correlation analysis results demonstrated that *Akkermansia* (*r* = −0.356, *P* = 0.024), *Desulfovibrio* (*r* = −0.316, *P* = 0.047), and *Alloprevotella* (*r* = −0.366, *P* = 0.020) were significantly negatively correlated with HDL-C. *Akkermansia* was also negatively correlated with LDL-C (*r* = −0.390, *P* = 0.012) and TP (*r* = −0.370, *P* = 0.019). In addition, *Desulfovibrio* was significantly positively correlated with glucose (GLU) (*r* = 0.352, *P* = 0.025) (Figure 5(c)). *Akkermansia* (*r* = 0.495, *P* = 0.026), *Odoribacter* (*r* = 0.467, *P* = 0.038), *Alistipes* (*r* = 0.579, *P* = 0.007), *Parabacteroides* (*r* = 0.522, *P* = 0.018), and *Parasutterella* (*r* = 0.465, *P* = 0.039) were positively correlated with NBD. *Akkermansia*, *Odoribacter*, and *Desulfovibrio* were also negatively correlated with BI, FMA-UE, and FMA-LE (*P* < 0.05). Both *Paraprevotella* and *Sutterella* were positively correlated with portions of the BPI (ADL and walking) (*P* < 0.05), and both *Akkermansia* (*r* = 0.605, *P* = 0.005) and *Odoribacter* (*r* = 0.471, *P* = 0.036) were positively correlated with VAS (Figure 5(d)).

## 4. Discussion

Several investigations have documented differences in the gut microbiome composition between poststroke patients and healthy subjects. In this study, we discovered that stroke patients had lower species diversity and evenness. The findings are consistent with the studies using rodent experimental stroke models [31]. Multiple studies have showed a considerable rise in the prevalence of Prevotella and a decrease in the prevalence of *Bacteroides* in stroke patients. We also observed a considerable reduction of *Bacteroides* in stroke patients, consistent with the study of Yin et al. [19]. *Bacteroides* play a leading role in the intestinal microbiota and were found to be associated with obesity [32, 33]. Furthermore, it has been found that a decrease in *Bacteroides* in cases of obesity and overweight is also recognized as one of the important risk factors for the ischemic stroke [34]. In addition, *Bacteroides* taxa have been shown to ferment polysaccharides to both acetate and propionate [35]. Previous studies have demonstrated a decreased relative abundance of *Akkermansia* in poststroke patients [36, 37]. In contrast, *Akkermansia* increased significantly after stroke in the current study. There has been a study indicating that an increase in the number of *Akkermansia* bacteria in the poststroke may facilitate the *Akkermansia*-assisted healing of wound damage and reinforce the epithelial integrity of the intestinal mucosa [38]. Meanwhile, some studies have shown greater abundance of *Akkermansia* in hypertensive subjects and it related to an overall proinflammatory environment, which is considered to be one of the mechanisms of stroke occurrence [39, 40]. Therefore, it is tempting to hypothesize that this microbiota member may have a role in stroke, and future research may uncover more unique activities of *Akkermansia*.

Our findings also revealed a decline in the amounts of fecal acetic acid and propionic acid in stroke patients. The most prevalent SCFAs are acetic, butyric, and propionic acids [41], and it appears that maintaining the function of the gut barrier involves a significant amount of SCFA generation [42]. Multiple mechanisms have been identified by which SCFAs affect the host, involving the control of acetylation and methylation of histones, the regulation of G-protein coupled receptors, the facilitation of the secretion of various hormones and neurochemicals, and the stimulation of signals through the vagus nerve [3]. SCFAs also serve as a source of energy in the mitochondria, which results in an exceptionally rapid absorption of these molecules in humans [43]. Acetic acid and propionic acid are the two primary metabolites that are produced by the microbiome of the gut, and they are responsible for regulating the actions of the microbiome-gut-brain axis. It has been demonstrated that certain concentrations of acetate and propionate exert a direct effect on the brain. The most frequent SCFA, acetate, is digested by the liver and subsequently transported to peripheral tissues, where it participates in cholesterol metabolism and lipogenesis and may have a role in the regulation of central appetite [44]. Acetate also acts as a fuel for the brain, and it easily penetrates through the blood–brain barrier from the periphery and is metabolized in the brain [45]. Previous research demonstrated that rats receiving fecal microbiota transplants from depressed patients showed increased fecal acetate and total SCFA concentrations as well as depression-like behavior [46]. According to Maltz et al., mice suffering from psychosocial stress exhibit a decrease in fecal acetate, which is accompanied by an increase in inflammation in the gut [47]. Additionally, the current study confirmed a negative association between fecal acetic acid and *Fusobacteria*, *Desulfovibrio*, and *Akkermansia*, which were significantly increased in poststroke patients. Propionate is the only SCFA that, after being digested, has the potential to be a significant source of glucose; it can be utilized for the production of energy and may have a role in decreasing cholesterol levels [48]. Some investigations have shown that propionate and butyrate can directly alter brain physiology and behavior by working on microglial cells and astrocytes to enhance anti-inflammatory activity and control general brain maintenance by scavenging damaged or unneeded neurons, synapses, and infectious agents [49, 50]. Collectively, our results and the aforementioned evidence indicate that acetate and propionate may govern the gut-brain axis in poststroke patients by modulating the immune system and energy metabolism.

Despite the fact that there was not a discernible change in the concentrations of caproic, valeric, isobutyric, isovaleric, or butyric acid between the two groups of our study, we found a negative correlation between *Fusobacteria* and butyrate, and *Fusobacteria* abundance was significantly higher in poststroke patients. There is evidence that butyrate stimulates vascular endothelial growth factor, which may play central roles in neurogenesis, angiogenesis, and functional recovery in the aftermath of stroke [51]. Furthermore, lower fecal butyrate concentrations were also associated with a high risk of stroke [52]. This might indicate that butyrate is involved in the progression of ischemic stroke. Isovaleric acid and isobutyric acid were negatively correlated with serum total protein, and valeric acid and caproic acid were negatively correlated with cholesterol. Isobutyrate, isovalerate, valerate, and caproate are generally considered the typical products of fat and protein fermentation, and they may have the ability to influence lipid metabolism, which affects the lipid profile of the host circulation in the disease state of stroke [35, 53]. These are the research directions warranting further investigation of these metabolites that have relatively low content.

According to the findings of our study, alterations in certain bacteria of the gut appear to be connected with improvements in pain, bowel function, ADL, and motor function of poststroke patients, prompting further investigation into the clinical impact of gut microbiota in this patient population. Some typical SCFA-producing bacteria, *Akkermansia* and *Odoribacter*, were found to be positively associated with VAS and NBD but negatively correlated with BI, FMA-UE, and FMA-LE. SCFAs are essential for intestinal barrier maintenance and microbial regulation [54]. Butyrate has a powerful anti-inflammatory effect on macrophages in the central nervous system, which can inhibit the inflammatory response, thus realizing the important role of nerve protection [55, 56]. Moreover, our current study is particularly concerned about chronic pain associated with stroke. Although SCFAs are crucial for regulating immune responses, their significance in neurological illnesses, particularly chronic pain, has just recently been recognized [57, 58]. SCFAs modulate the production of inflammatory mediators by macrophages, which is mainly associated with the attenuation of histone deacetylase (HDAC) activity and is able to attenuate pain behaviors [59, 60]. In a rat permanent middle cerebral artery occlusion model, valproic acid and butyrate, as HDAC inhibitors, presented antineuroinflammatory and neuroprotective effects after stroke [61]. This suggested that SCFAs may play a significant role as key mediators in the modulation of pain in poststroke patients. However, the mechanism underlying this phenomenon is not singular; there may be multiple mechanisms that influence each other and promote each other to ultimately achieve functional recovery.

Despite its innovative findings and clinical relevance, the present study included a number of limitations. Larger samples and multicenter studies would be required for further validation of the findings because the study was restricted to just one center, with a somewhat small patient enrollment. Then, the study investigated the changes in microbiota and SCFA levels following stroke and showed that there may be links between changes in the gut microbiota and clinical functional parameters. However, the scope of our clinical indicators is limited and needs to be further expanded and more needs to be done to adjust for the effects from the risk factors of stroke including dysglycemia and dyslipidemia. Further studies focusing on possible biological mechanisms are needed. Finally, grading for the severity of stroke in terms of mild, moderate, and severe was not performed, so the correlation between the severity of disease and gut microbiota could not be analyzed. This will be addressed specifically in future experiments.

## 5. Conclusion

In conclusion, a shift in the gut microbiota and its connection with fecal SCFAs was identified in poststroke patients in comparison to healthy controls. Significant associations were detected between alterations in SCFA levels, as well as distinctive gut microbiota and poststroke clinical outcomes or functional prognosis. Treatment strategies aimed at modulating the gut microbiota and SCFAs may have the potential to relieve pain and improve the functional prognosis after stroke.

## Figures and Tables

**Table 1:** Characteristics of study participants.

	Poststroke group (*n* = 20)	Control group (*n* = 20)	*P* value
Age in year, mean (SD)	63.55 (12.63)	59.95 (8.02)	0.290
Gender, *n* (%)			0.201
Male	11 (55.00)	6 (30.00)	
Female	9 (45.00)	14 (70.00)	
Height in centimeter, mean (SD)	167.55 (6.19)	163.50 (6.49)	0.069
Weight in kilogram, mean (SD)	66.80 (8.03)	62.90 (5.53)	0.238
BMI in kg/m^2^, mean (SD)	23.79 (2.57)	23.47 (2.01)	0.265
SBP in mmHg, mean (SD)	127.10 (20.45)	118.10 (16.82)	0.390
DBP in mmHg, mean (SD)	78.35 (10.33)	76.00 (8.37)	0.434
Smoking status, *n* (%)			0.723
Nonsmoker	7 (35.00)	9 (45.00)	
Current smoker	7 (35.00)	7 (35.00)	
Previous smoker	6 (30.00)	4 (20.00)	
Alcohol intake, *n* (%)			0.326
No drinking	7 (35.00)	12 (60.00)	
Light drinking	8 (40.00)	6 (30.00)	
Heavy drinking	5 (25.00)	2 (10.00)	
Medical history, *n* (%)			
Hypertension	15 (75.00)	9 (45.00)	0.053
Diabetes mellitus	9 (45.00)	2 (10.00)	0.013
Dyslipidemia	7 (35.00)	2 (10.00)	0.058
Laboratory findings			
Total protein (g/L)	62.00 (5.00)	64.81 (6.04)	0.118
Albumin (g/L)	39.48 (3.28)	39.99 (3.07)	0.615
Total bilirubin (*μ*mol/L)	12.15 (3.01)	14.63 (6.59)	0.134
Direct bilirubin (*μ*mol/L)	2.00 (0.76)	2.04 (0.96)	0.500
ALT (U/L)	24.55 (17.18)	22.05 (14.09)	0.425
AST (U/L)	24.30 (9.38)	24.45 (9.09)	0.919
Urea (mmol/L)	6.17 (1.86)	5.86 (2.88)	0.689
Creatinine (*μ*mol/L)	68.17 (22.38)	76.66 (38.83)	0.403
Uric acid (*μ*mol/L)	290.44 (116.44)	278.10 (103.76)	0.726
Glucose (mmol/L)	6.63 (1.69)	5.62 (1.19)	0.035
Cholesterol (mmol/L)	4.67 (1.14)	5.05 (1.08)	0.280
Triglyceride (mmol/L)	1.58 (0.76)	1.50 (0.62)	0.654
HDL-C (mmol/L)	0.99 (0.29)	1.06 (0.15)	0.329
LDL-C (mmol/L)	2.07 (0.83)	2.27 (0.45)	0.359
Stroke characteristics			
Type of stroke, *n* (%)			
Hemorrhage stroke	7 (35.00)		
Ischemic stroke	7 (65.00)		
Duration of stroke, *n* (%)			
No more than 3 months	11 (55.00)		
More than 3 months	9 (45.00)		
Side of hemiparesis, *n* (%)			
Left	8 (40.00)		
Right	12 (60.00)		
FMA-UE score, mean (SD)	15.8 (10.94)		
FMA-LE score, mean (SD)	16.30 (6.07)		
Barthel index score, mean (SD)	43.00 (17.73)		
VAS score, mean (SD)	4.55 (1.36)		
NBD score, mean (SD)	14.55 (5.38)		
BPI score, mean (SD)			
Activity of daily living	4.75 (1.52)		
Emotion	4.80 (1.40)		
Sleep	3.85 (1.35)		
Work	4.55 (1.43)		
Walk	4.90 (1.65)		
Relationship	4.95 (0.89)		
Interests	5.85 (1.09)		

SD: standard deviation; BMI: body mass index; SBP: systolic blood pressure; DBP: diastolic blood pressure; ALT: alanine aminotransferase; AST: aspartate aminotransferase; HDL-C: high-density lipoprotein cholesterol; LDL-C: low-density lipoprotein cholesterol; FMA-UE: Fugl-Meyer Assessment Upper Extremity Scale; FMA-LE: Fugl-Meyer Assessment Lower Extremity Scale; VAS: Visual Analogue Scale; NBD: Neurogenic Bowel Dysfunction; BPI: Brief Pain Inventory.

## Data Availability

The data used to support the findings of this study are available from the corresponding authors upon request.
